# Supplementary material for: The interactome of intact mitochondria by cross-linking mass spectrometry provides evidence for coexisting respiratory supercomplexes
Source: Mol Cell Proteomics. 2017 Dec 8;17(2):216–32. doi: 10.1074/mcp.RA117.000470 (PMC5795388; doi:10.1074/mcp.RA117.000470)
Supplement: Supplemental Data [file supp_RA117.000470_133922_0_supp_23978_fzffwf.zip › spectra_annotation/mito_DR_spectra_annotation/479-1-10-1-6-1.pdf]

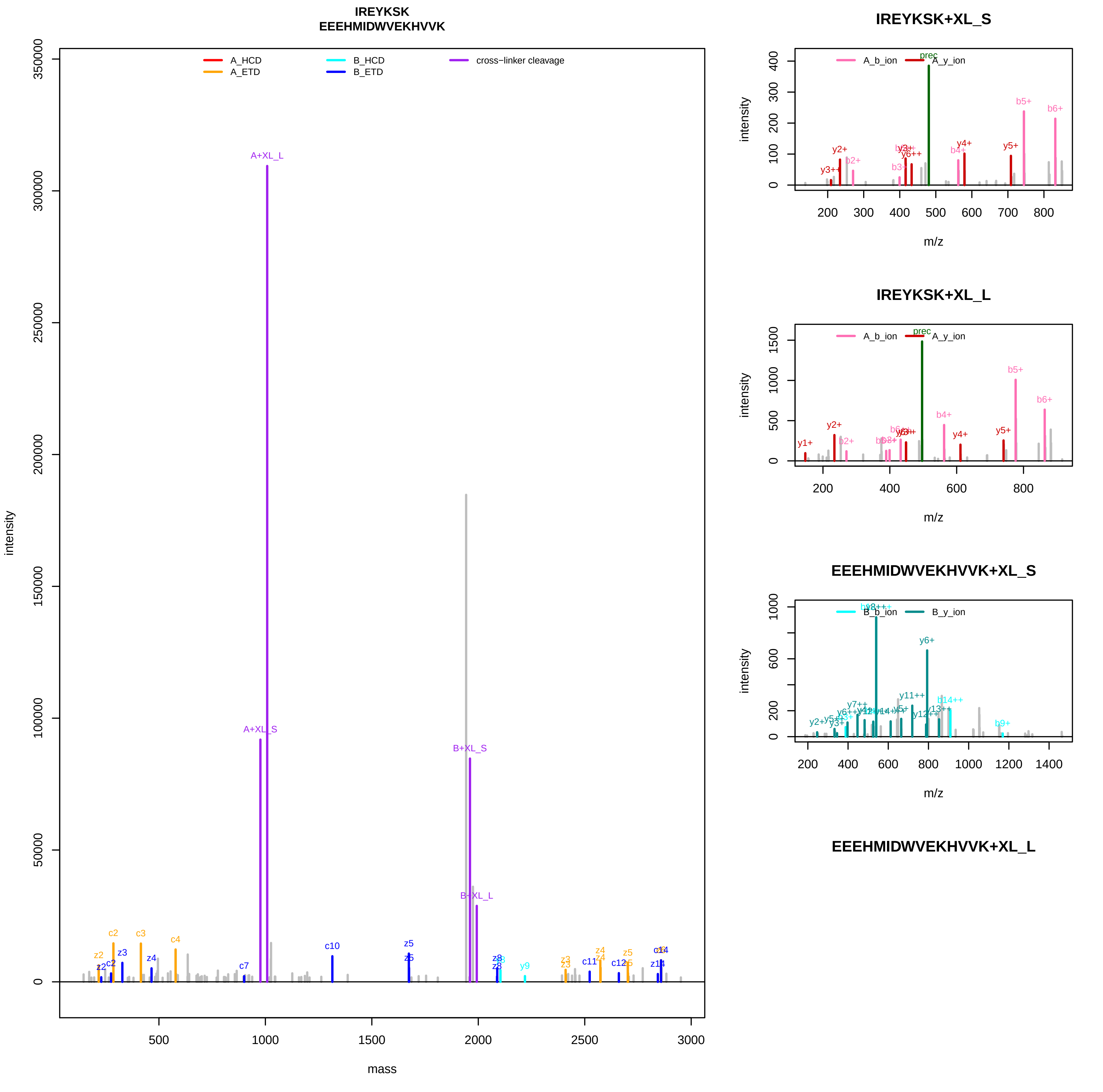

IREYKSK
EEEHMIDWVEKHVVK
A_HCD
A_ETD
B_HCD
B_ETD
cross−linker cleavage
intensity
mass
0
50000
100000
150000
200000
250000
300000
350000
500
1000
1500
2000
2500
3000
A+XL_L
A+XL_S
B+XL_S
B+XL_L
c2
c3
c4
z2
z3
z4
c7
c10
z5
z8
y9
c11
c12
z14
c14
IREYKSK+XL_S
A_b_ion
A_y_ion
prec
y2+
y3++
b2+
b3+
b4+
y4+
y5+
b5+
b6+
y6++
m/z
IREYKSK+XL_L
y1+
EEEHMIDWVEKHVVK+XL_S
B_b_ion
B_y_ion
y6+
y11++
y12++
y13++
b14++
b9+
y2+
y3+
y7++
EEEHMIDWVEKHVVK+XL_L
